# *Plant Physiology* is recruiting Assistant Features Editors to start in 2025 and 2026

**DOI:** 10.1093/plphys/kiae304

**Published:** 2024-06-12

**Authors:** Yunde Zhao, Mary Williams

**Affiliations:** Cell and Developmental Biology, University of California San Diego, La Jolla, CA 92093-0116, USA; Features Editor, Plant Physiology, ASPB, USA

We started the *Plant Physiology* Assistant Features Editor program more than 6 years ago to help young scientists to advance their career and help disseminate exciting discoveries published in *Plant Physiology*. Since then, more than 100 early-career scientists, mostly postdocs but also some advanced graduate students, have served as Assistant Features Editors (AFEs).

This past January, *Plant Physiology* welcomed 15 new AFEs to the editorial board. Together with the AFEs recruited in 2023, these young scientists have brought their passion for science to the journal, communicating to our readers some of the most exciting *Plant Physiology* papers through News and Views articles. Some examples of News and Views written by AFEs can be found here https://academic.oup.com/plphys/issue#1873624-7517351.

Our AFE program greatly advances young scientists’ careers; in fact, of those who have completed the program and moved into their next roles, the majority have secured tenure-track positions. *Plant Physiology* AFEs receive professional advice on scientific writing from our seasoned editors, and the AFEs have abundant opportunities to network with our regular editors. For example, AFEs conduct informal interviews with *Plant Physiology* editorial board members, enabling the AFEs and *Plant Physiology* readers to get to know our editors (see https://plantae.org/research/plant-physiology/#editor-profiles). AFEs are invited to the *Plant Physiology* editorial board meetings, and for the in-person meeting, their travel expenses are covered by the journal. At the editorial board meetings, the AFEs not only can interact with and learn from senior scientists but also contribute to discussions on journal policies and operation.

One of our AFEs recently shared, “I really enjoy writing News and Views and can already see improvement in my writing style!” and another wrote, “The editorial meeting last year was great. It was very interesting to learn about topics of concern for this journal and hear the discussions to try and mitigate them. The AFE program is amazing and I am very grateful to be part of it.”

In the spirit of cooperation, *Plant Physiology* and *Plant Cell* are now recruiting AFEs in alternating years; this year, *Plant Physiology* is recruiting AFEs to start in 2025 and 2026, and next year, *Plant Cell* will recruit AFEs to start in 2026 and 2027. Therefore, we are currently recruiting two cohorts of AFEs to start in either January 2025 or January 2026. Each new cohort will work with the journal for 24 months.

**If you are interested in becoming an Assistant Features Editor, we are welcoming applications through Tuesday, October 15, 2024.** Further details of the activities of the AFEs and how to apply are available online at https://blog.aspb.org/plant-physiology-is-recruiting-assistant-features-editors-to-start-in-2025-and-2026/.

We are interested in hearing about your current position and future goals and why you are interested in the position. We will need your Curriculum Vitae and the contact details of two individuals familiar with your work as a researcher. We will also expect two samples of your writing, one a first-authored paper and the other a sample News and Views commentary you write on your selection of one of the *Plant Physiology* articles listed at https://blog.aspb.org/plant-physiology-is-recruiting-assistant-features-editors-to-start-in-2025-and-2026/. We hope to have selected the new AFEs in late November.

